# Temporomandibular joint function, periodontal health, and oral microbiome in early rheumatoid arthritis and at-risk individuals: a prospective cohort study protocol

**DOI:** 10.1038/s41405-020-0034-8

**Published:** 2020-05-19

**Authors:** J. M. Kroese, C.M.C. Volgenant, D. van Schaardenburg, B. G. Loos, W. Crielaard, F. Lobbezoo

**Affiliations:** 1 Department of Orofacial pain and Dysfunction, Academic Centre for Dentistry of Amsterdam (ACTA), University of Amsterdam and Vrije Universiteit Amsterdam, Amsterdam, The Netherlands; 2 Department of Preventive Dentistry, Academic Centre for Dentistry of Amsterdam (ACTA), University of Amsterdam and Vrije Universiteit Amsterdam, Amsterdam, The Netherlands; 3 Department of Periodontology, Academic Centre for Dentistry of Amsterdam (ACTA), University of Amsterdam and Vrije Universiteit Amsterdam, Amsterdam, The Netherlands; 4 Amsterdam Rheumatology and Immunology Center, Reade and Amsterdam University Medical Center, Amsterdam, The Netherlands

**Keywords:** Periodontitis, Mandibular muscles, Dental biofilms, Periodontitis, Temporomandibular disorders

## Abstract

**Objectives/aims:**

Rheumatoid arthritis (RA) is an autoimmune disease affecting the joints, including the temporomandibular joint (TMJ). Early diagnosis and treatment can alleviate symptoms and prevent progression. Predictors for disease outcome in individuals at risk for RA are therefore valuable. While limited information is available on the prevalence of TMJ involvement in early RA, previous studies suggest that RA, periodontitis and the oral microbiome are interrelated. Predictive factors for RA development may thus be present in the oral cavity. Our two aims are: (1) to assess the prevalence of TMJ involvement in early RA, and (2) to investigate the predictive value of oral factors in RA development.

**Materials and methods:**

We will include 150 individuals in this multi-center, prospective cohort study: 50 patients with early RA, 50 at-risk individuals, and 50 healthy controls. At baseline, the TMJ, periodontal health, and the oral microbiome will be examined. The general health will be followed over time, on four occasions up to 3 years.

**Discussion:**

Our results will provide insight into the prevalence and clinical characterization of TMJ involvement in early RA. For at-risk individuals, oral factors can be studied as possible predictors for the development of RA.

## Background

Rheumatoid arthritis (RA) is an autoimmune disease affecting the synovial joints that eventually results in the destruction of cartilage and bone.^1^ In developed countries, RA affects 0.5–1.0% of the adult population. Because no cure is available for RA, pharmacological treatment focuses on arresting disease progression and alleviating symptoms.^2^ Early intervention results in better clinical outcomes^3^ and may prevent joint destruction,^4^ which makes early diagnosis desirable.

Most RA patients are affected by the so-called seropositive form of RA, defined by the presence of specific antibodies: IgM rheumatoid factor (IgM-RF) and antibodies against citrullinated proteins (ACPA).^5^ Increased serum levels of these antibodies in healthy individuals imply an elevated risk of developing RA.^6^ Interestingly though, not everyone having increased IgM-RF or ACPA serum levels develops RA. Since early diagnosis of RA is favorable, additional predictors of RA development in at-risk individuals would be valuable. Prediction models based on clinical, serological, and imaging variables have already been developed.^7,8^

Additional predictors might be present in the orofacial tissues; RA can affect the temporomandibular joint (TMJ), and previous studies suggest that RA, periodontitis, and the oral microbiome are interrelated, as outlined in detail below.

## RA and the TMJ

Being a disease affecting the joints, RA can also affect the TMJ, causing disease-related symptoms.^9–11^ A prevalence of TMJ disorders (TMD) in patients with RA ranging from 19% to 85.7% has been reported.^12–14^ The wide range in prevalence is presumably caused by variations in diagnostic criteria and assessment methods. For patients with early RA, only limited data is available on TMD prevalence; based only on pain on palpation, a prevalence of 10.6% has been reported.^15^ To our knowledge, no data on TMD is available for individuals with an increased risk of developing RA. Therefore, research on these specific patient groups is needed.

## RA and oral health

Reduced saliva production is more prevalent in patients with RA than in people without RA.^9^ Furthermore, a positive association between RA and periodontitis has been described,^16,17^ although other studies did not find this relation.^18–20^ The possible role of *Porphyromonas gingivalis*—a bacterium highly associated with periodontitis—in the immunopathogenesis of RA has been of particular interest due to its capacity to generate citrullinated proteins.^5,21^

An interesting development in research on microbiology is to focus on the microbiome as a whole, grouping species based on their 16 S rDNA gene, instead of focusing on specific bacteria.^22^ Therefore, it is of importance to investigate the oral microbiome in relation to RA, and the possible association between oral microbiome dysbiosis and disease progression. Of particular interest are individuals at increased risk of developing RA, and the possible biomarkers in the oral microbiome for RA development.

## Aim and objectives

The overall aim of the study described in this protocol is to investigate several aspects of the interaction between RA and the orofacial tissues, focusing on patients with early RA and individuals with an increased risk of developing RA.

The primary objective is to determine the prevalence of TMD within these two groups and a healthy control group. The secondary objectives are to investigate the possible predictive value of oral health and oral microbiome on the development and progression of RA, and to explore the dynamics of the microbiological composition of the oral ecosystem of the three groups.

For the primary objective, the hypothesis is that the prevalence of TMD is higher in patients with early RA and patients with an increased risk of developing RA, compared to a healthy control group. For the secondary objectives, the hypotheses are that periodontal inflammation and oral microbiome dysbiosis might contribute to the development of RA and negatively influence the progression of RA, and that the microbiological composition of the oral ecosystem differs between the three groups.

## Methods and design

### Study design

The study has a multi-center, prospective cohort study design. Participants will be recruited in three different groups; (1) patients who have been diagnosed with early RA, (2) persons with an increased risk of developing RA, and (3) a control group with no autoimmune conditions. A total number of 150 subjects will be recruited, i.e., 50 in each group. Groups 1 and 2 will be recruited at Reade, an out-patient clinic for rheumatology in Amsterdam, while group 3 will be recruited at ACTA, the Academic Center for Dentistry Amsterdam.

Extensive research data will be gathered at baseline, and all examinations will take place at Reade. The development of general health will be followed up over time, on four occasions up to three years after baseline. The length of the follow-up period was determined based on a study on individuals at-risk of developing RA performed at Reade. This study showed that those who developed arthritis, did so after a median (IQR) follow-up of 12 (6–23) months, and the descending curve of arthritis-free survival flattened after a follow-up of 36 months.^7^

### Participants

Subjects are eligible for inclusion if they are aged 18 years or older, have a minimum of 12 natural teeth, and are willing and able to give written informed consent in the Dutch language.

For group 1, patients should be diagnosed with RA according to the treating rheumatologist, within the last year, based on the 2010 EULAR RA criteria.^23^ For group 2, the increased risk of developing RA has to be determined by increased serum levels of IgM-RF and/or ACPA.

For group 3, healthy subjects will be recruited at ACTA. Potential subjects for the control group will be excluded when they have an autoimmune condition, or when increased serum levels of IgM-RF or ACPA are found during evaluation of a blood sample collected at the baseline visit. Also, students from ACTA and employees of ACTA and Reade will be excluded from participation in this study, to prevent bias caused by above average oral hygiene/self-care in health care providers.

All potential subjects at Reade will be invited by the treating doctor or nurse to participate in the study. After oral consent, they are contacted and thoroughly informed about the study by a dedicated investigator (JMK). Recruitment for the control group at ACTA will start approximately 6 months after the first inclusion at Reade, to be able to match the control group to the groups at Reade for sex and age. Potential subjects at ACTA will be approached and thoroughly informed by the dentist-investigator (JMK). Subjects in all three groups receive at least 1 week time to consider participation after being thoroughly informed. If a subject is willing to participate, written consent is obtained.

For all study subjects, participation is always voluntary, and declining to participate will not be of any influence on the medical or dental care patients receive at either Reade or ACTA.

### Sample size

The primary objective of this study—to assess the prevalence of TMD in patients with early RA or with an increased risk of developing RA—was used to determine the sample size.

Previous studies have reported a prevalence of TMJ pain in patients with RA ranging between 19% and 85.7%.^12–14^ However, because of the wide variations on disease duration, these studies do not provide specific information on prevalence in patients with early RA. A recent study conducted at Reade on TMJ pain in patients with early RA, using pain on palpation as the only outcome measure, reported a prevalence of 10.6%.^15^ To our knowledge, no prevalence data is available on individuals at increased risk of developing RA. In comparison, for the general Dutch population a prevalence of TMD from 5%^24^ to 8%^25^ has been reported. The lack of sufficient data on patients with early RA or an increased risk of developing RA having TMD, complicates an accurate assessment of the required group sizes. Furthermore, we anticipate different outcomes on prevalence of TMD compared to previous studies, because of the more extensive examination of the masticatory system as compared to previous studies, taking into account both arthrogenous and myogenous components, as further described below.

The limited availability of data complicates a reliable sample size calculation. Thus, based on expectations and anticipated feasibility to recruit participants, we estimate that 30-40 subjects per group are required to adequately explore the primary objective and to determine whether it would be realistic to plan a larger (multi-center) study. Because of an anticipated drop-out rate of 20% after three years, the aim is to include 50 subjects in each group.

### Outcome variables

#### TMJ disorders

A TMD diagnosis will be determined according to the Diagnostic Criteria for Temporomandibular Disorders (DC/TMD).^26^ The combination of a questionnaire and standardized clinical tests is necessary to determine the possible diagnosis or diagnoses.

Prior to the baseline visit, participants are asked to fill in the symptom questionnaire on TMD pain, headache, jaw joint noises, and closed and open locking of the jaw. During the baseline visit, a standardized examination of the TMJs and the masticatory muscles (temporalis and masseter) will be performed, as described in the DC/TMD clinical examination protocol.^27^ Tests will be limited to those items of the protocol needed to be able to determine a TMD diagnosis.

It has been reported that dynamic joint tests and static muscle tests can be of additional value for the confirmation of a suspicion on TMD pain. These tests, distinguishing both an arthrogenous and a myogenous component of TMD, result in a higher specificity than palpation alone.^28^ Therefore, in addition to the DC/TMD clinical examination protocol tests, which mainly exist of palpation tests, the dynamic and static tests will be performed.

The dentist performing the clinical research (JMK) was trained in performing the described TMJ examinations by a calibrated specialist in TMD.

#### General health

Each participant will be asked to fill in a standard medical questionnaire prior to the baseline visit, to be able to track general health and identify possible confounders, such as comorbid conditions and medication use. During the baseline visit, additional questions will be asked about recent use of antibiotics, because of their reported effect on the oral microbiome,^29^ and of analgesics, because of the potential masking effect on pain during the performance of the TMD tests and their possible anti-inflammatory characteristics affecting periodontal tissues and microbiome.

Venous blood is collected from all participants in groups 1 and 2 at intake as a standard procedure at Reade, so analyses from these samples can be used for the current study. To determine serum levels of IgM-RF and ACPA in the control group, a blood sample will be collected during the baseline visit.

On four occasions up to three years after baseline, the patient files of participants will be consulted to determine possible changes in the general health. If information in a patient file is insufficient, the participant will be contacted by phone to collect complementary data.

#### Oral health

At baseline, the oral condition of all participants is measured for several intra-oral aspects, but particularly the periodontal health by performing a full periodontal examination. Presence or absence of bleeding on probing (BOP), gingival recessions (positive and negative, to also identify pseudopockets) in millimeters, and pocket probing depth (PPD) in millimeters will be measured on six sites for each tooth (mesiobuccal, midbuccal, distobuccal, mesiolingual, midlingual, and distolingual). Recording of BOP results in a full mouth BOP percentage. The combination of recessions and PPD results in the amount of clinical attachment loss (CAL). Prior to the probing, the amount of dental plaque will be scored according to the Silness-Loë Index.^30^ (on a scale from 0 to 3, with 0 being “no plaque” and 3 “abundance of soft matter within the gingival pocket and/or on the tooth and gingival margin”) for all teeth on all six previously described sites. The dentist-examiner (JMK) was trained for the periodontal examination by a periodontologist.

In addition to the examination of periodontal health, a visual intra-oral inspection will be performed to record possible mucosal lesions and the amount of decayed, missing, and filled surfaces of the teeth (DMFS).^31^ The main utility of this inspection is the exclusion of possible sources of intra-oral pain as a confounding factor. In case periodontitis and/or overt caries will be found, the participant and the treating dentist will be informed.

#### Bruxism

At baseline, both subjective and objective bruxism activity will be measured to study a possible mediating role of bruxism in the prevalence and characterization of TMD in the study population. Subjective bruxism is determined by asking the participants if they believe they perform bruxism activity, and if so in what frequency. A distinction between awake bruxism and sleep bruxism will be made.^32^

Objective bruxism is determined based on several intra-oral aspects, i.e., the presence or absence of a bruxoposition (the occlusal contact position to which one grinds and in which one clenches), impressions of the soft tissues (cheeks, tongue, and/or lips), the suspected nature—being mechanical, chemical, or both—of existing tooth wear, and quantification of existing tooth wear. The quantification of tooth wear is recorded using the screening module of the comprehensive Tooth Wear Evaluation System,^33^ resulting in a score of 0 to 4, with 0 being “no (visible) wear” and 4 being “loss of clinical crown height >2/3”, for occlusal/incisal wear for each sextant,^34^ and a score of 0 to 2, with 0 being “no wear” and 2 being “wear with dentine exposed”, for wear on the palatinal surfaces of the second sextant.

Findings will be used to determine a bruxism diagnosis according to the diagnostic grading system proposed by Lobbezoo et. al.,^32,35^ where “possible” sleep or awake bruxism will be diagnosed based on self-report, and ‘probable’ sleep or awake bruxism will be diagnosed based on the combination of self-report and clinical findings.

#### Oral microbiome

During the baseline visit, samples for oral microbiome analyses will be collected. The oral microbiome has been reported to significantly differ between different oral niches,^36^ and therefore a variety of samples will be collected. Subgingival dental plaque will be collected from the first molar of the fourth quadrant (when needed, the other molars and premolars will be used in a standardized order), using a sterile universal curette. Participants will be asked to chew paraffin to enable the collection of a stimulated saliva sample. A sample of the tongue coating will be collected by swiping a microbrush on the dorsum of the tongue.

The microbiome of these samples will be assessed to determine a possible presence and possible role of a dysbiosis in the development of RA. Based on the 16 S rDNA, species will be grouped in Operational Taxonomic Units (OTU’s), after which samples can be grouped in clusters based on similarities using Neighborhood Co-regularized Spectral Clustering (NCSC), an approach used in previous microbial ecological studies.^37,38^ These clusters will be used as the microbiological outcome value for further analyses.

In addition to the collection of samples, the fluorescence of dental plaque will be studied.^39^ Participants will be asked to refrain from oral hygiene (i.e., brushing the teeth, using interdental devices, mouth wash, and chewing gum) 24 h prior to their baseline visit. During the baseline visit, a total of six photographs of the dentition will be taken—three fluorescence photographs, and three corresponding white light photographs. To be able to explore a possible influence of nutrition on plaque fluorescence, all participants will be asked to keep a food diary during the three days prior to their baseline visit.

#### Intra-oral immuno-biochemical characteristics

Gingival Crevicular Fluid (GCF), containing cells related to the immune system and therefore portraying immune-biochemical characteristics, will be collected at baseline.^40^ GCF will be collected at four sites—the mesial sides of the upper canines and the distal sides of the upper central incisors—by means of a sterile paper strip at the entrance of the crevice. The paper strip will be held in place during 30 seconds and a new paper strip will be used for each site.

Quantification of the absorbed GCF will be determined immediately after removing the paper strip, by using a same-day calibrated Periotron 8000 (Oraflow Inc., New York, NY, USA). Because local conditions have been described to influence outcome values of the Periotron 8000,^41^ both temperature and humidity will be recorded during calibration and GCF collection to be able to detect possible substantial differences between locations or over time.

#### Oral health-related quality of life

Prior to the baseline visit, participants will be asked to fill in the shortened Oral Health Impact Profile questionnaire (OHIP-14), derived from the original 49-item OHIP, a tool for assessing the social impacts of oral disorders.^42^ With the OHIP-14, the frequency of a variety of possible impacts (14 questions) during the past month is scored on a five-point ordinal scale, ranging from 0 (“never”) to 4 (“very often”), resulting in a total score ranging from 0 to 56. A Dutch translation of the OHIP-49 has been developed and validated,^43^ and the shorted OHIP-14 has been reported adequate for replacing the original 49-item OHIP.^44^

#### Self-reported disease status

At Reade, patients’ self-reported physical function, pain, and global estimate of disease status are periodically measured by the RAPID-3 questionnaire—a Routine Assessment of Patient Index Data described to be valuable for measuring disease status.^45^ If recent RAPID-3 data for a participant is available at Reade, this data will be used for the current study. If no recent data is available, participants will be asked to fill in the RAPID-3 questionnaire prior to their baseline visit. This also applies to participants in the control group.

The first section of the RAPID-3, physical function, is scored for several activities on a scale from 0 (“without any difficulty”) to 3 (“unable to do”), converted to a total score ranging from 0 to 10. The second (pain) and third (global estimate of disease status) sections of the questionnaire are scored on a scale from 0 (“no pain”/”very well” respectively) to 10 (“pain as bad as it could be”/”very poorly” respectively). The RAPID-3 results in an overall score ranging from 0 to 30.

#### Xerostomia

To measure symptoms of xerostomia, participants will be asked to fill in the Xerostomia Inventory questionnaire^46^ prior to the baseline visit. The questionnaire contains 11 questions about the frequency in which someone had to act on, or had trouble functioning because of, the adverse consequences of xerostomia during the past four weeks, scored on a scale from 1 (“never”) to 5 (“very often”).

### Statistical analysis

#### Descriptive statistics

Characteristics of the study cohort will be reported. For continuous variables, the mean, median, standard deviation, minimum and maximum values (or the range) will be reported, together with a percentage of frequency values.

#### Analyses of study parameters

The differences between groups in the presence of a defined TMD diagnosis will be analyzed using the Chi-square test. Differences in outcomes of the several separate DC/TMD tests—not necessarily resulting in a defined diagnosis—will be analyzed with the Friedman test (one-way repeated measures analysis of variance by ranks). For the longitudinal outcomes as well as some of the secondary outcomes, a Generalized Estimation Equation analysis will be used. A two-sided alpha level of 0.05 will be used for all statistical analysis.

An interim analysis will be performed when ~20 subjects in each group have been included. Cumulative frequency analysis will be used to predict future outcomes, and to determine if including the planned amount of subjects will add value to the existing results. By performing an interim analysis, possible unnecessary burden for the (future) participants can be prevented.

### Ethics approval and consent to participate

The study protocol has been approved by the accredited Medical Ethical Committee of the Slotervaart Hospital and Reade (*METc Slotervaartziekenhuis en Reade*, U/17.056/P1719) according to the Declaration of Helsinki (2013) and in accordance with the Medical Research Involving Human Subjects Act (WMO). The study has been included in the general assessment and registration form (ABR form, NL61521.048.17) and the Dutch National Trial Register (NTR, NL6198). Written informed consent is obtained from all study participants.

## Discussion

This research proposal for a prospective cohort study is intended to investigate the orofacial tissues—including the TMJ, oral health, and the oral microbiome—in patients with early RA and those at increased risk of developing RA. Although the strengths of this study protocol lie in extensive collection of research data at baseline and the longitudinal design, some limitations should also be mentioned.

Insufficient data is available to accurately calculate the group sizes that are required to explore TMD prevalence in the various groups. This makes the study design challenging. However, by using comprehensive tests to examine the masticatory system, distinguishing both arthrogenous and myogenous components, and by focusing on two very specific patient groups, we believe this study could provide a valuable addition to the limited data currently available. If our results indicate a difference in prevalence between groups 1 and 2 and the control group, a larger validation study could be planned to further explore this topic. Although Reade is seen as the RA expertise center and the referral clinic for a large area in the western Netherlands, the number of people at increased risk of RA who are being referred is still limited. A future multi-center study, possibly international, might therefore be needed to include a larger number of subjects as to increase the generalizability.

With regard to groups 1 and 2, our dependence on the patient intakes at Reade will not enable us to achieve one-to-one matching on age and sex for these two groups. However, we anticipate that subjects in group 1 and 2 will not differ significantly in age or sex distribution, as the increased risk of developing RA is a possible precursor of early RA. To approach accurate matching of the control group, recruitment of this group will start with a few months delay, aiming for a control group with an average age and sex distribution that does not statistically differ from the two other groups.

It is known that medication can significantly influence oral health and the oral microbiome,^29,47^ and as the effects of anti-rheumatic agents have been described specifically,^48^ patients’ medication is an important factor that may create a wide variety within group 1. As we expect that basing subgroups on medication use will greatly fragment group 1, we will analyze the results for this group as a whole, considering medication as a potential confounding factor.

As for the determination of a TMD diagnosis, there is a minor limitation. If after clinical examination a diagnosis is still uncertain, the DC/TMD describes ‘imaging’ by MRI or CT as the final step for confirming certain diagnoses (disc displacement and degenerative joint disease).^49^ However, imaging is not feasible within the scope of this study. On the other hand, it has also been suggested that imaging should be considered only if the information it provides would influence patient care.^50^ Therefore, since this study will not indicate any treatment, there is no basis for imaging. A similar limitation applies to the ‘definite’ diagnosis of bruxism: as no polysomnographic recording or electromyographic recording will be performed, the diagnosis will be limited to ‘possible’ or ‘probable’ sleep or awake bruxism according to the diagnostic grading system for bruxism.^32^

Despite these limitations, we argue that the outcomes of this study will provide new and important insight into a wide variety of factors regarding the interaction between RA and the orofacial tissues. It may thus indicate a direction for further research in this area.
